# Effects of a multi‐domain intervention against cognitive decline on dementia risk profiles – Results from the AgeWell.de‐trial

**DOI:** 10.1002/alz.085326

**Published:** 2025-01-09

**Authors:** Andrea Zülke, Alexander Pabst, Melanie Luppa, David Czock, Birgitt Wiese, Wolfgang Hoffmann, Thomas Frese, Jochen Gensichen, Hans‐Helmut König, Hanna Kaduszkiewicz, Jochen René Thyrian, Steffi G. Riedel‐Heller

**Affiliations:** ^1^ Institute of Social Medicine, Occupational Health and Public Health (ISAP), Medical Faculty, University of Leipzig, Leipzig, Saxony Germany; ^2^ Heidelberg University, Heidelberg, Hesse Germany; ^3^ Institute for General Practice, Hannover Medical School, Hannover, Lower Saxony Germany; ^4^ Institute for Community Medicine / University of Greifswald, Greifswald Germany; ^5^ Deutsches Zentrum für Neurodegenerative Erkrankungen e.V. (DZNE) and Institute for Community Medicine / University of Greifswald, Greifswald, Mecklenburg Western Pomerania Germany; ^6^ Institute of General Practice and Family Medicine, Martin‐Luther‐University Halle‐Wittenberg, Halle, Saxony‐Anhalt Germany; ^7^ Klinikum der Ludwig‐Maximilians‐Universität München, München, Bavaria Germany; ^8^ University Medical Center Hamburg‐Eppendorf, Hamburg, Hamburg Germany; ^9^ University of Kiel, Kiel, Schleswig‐Holstein Germany; ^10^ Deutsches Zentrum für Neurodegenerative Erkrankungen e.V. (DZNE) and Institute for Community Medicine / University of Greifswald, Greifswald Germany

## Abstract

**Background:**

Dementia risk scores have been suggested a promising surrogate outcome for lifestyle interventions targeting cognitive function and dementia risk. First evidence suggests beneficial effects of multidomain lifestyle interventions on dementia risk scores. We investigated effects of the multidomain AgeWell.de‐intervention on dementia risk, assessed using the LIfestyle for BRAin health (LIBRA)‐index.

**Method:**

Secondary analyses of AgeWell.de, a multicomponent intervention (including optimization of nutrition, medication, physical, social and cognitive activity) in older adults at increased risk for dementia (trial registration: German Clinical Trials Register, DRKS; ID: DRKS00013555). We analyzed data from n = 461 participants (age: 60‐77 years) with available information on all n = 12 risk/protective factors comprised by the LIBRA (coronary heart disease, diabetes, hypercholesterolemia, hypertension, depression, obesity, smoking, physical inactivity, renal disease, low‐to‐moderate alcohol use, high cognitive activity, healthy diet) at baseline and 24 months‐follow‐up. Intervention effects on LIBRA‐scores and individual LIBRA‐components were assessed using generalized linear models.

**Result:**

The intervention reduced total LIBRA‐scores, indicating a decreased risk for dementia at 24 months follow‐up (b = ‐0.63, 95% CI: ‐1.14, ‐0.12). Intervention effects on LIBRA‐scores were particularly due to favorable changes in diet (OR = 1.60, 95% CI: 1.16, 2.22) and hypertension (OR = 1.61, 95% CI: 1.19, 2.18). In younger participants (60‐69 years), the intervention increased the odds of high cognitive activity at follow‐up (OR = 2.00; 95% CI: 1.20, 3.34).

**Conclusion:**

The AgeWell.de‐intervention successfully reduced dementia risk, assessed using the LIBRA‐score, underscoring the usefulness of the LIBRA as a surrogate outcome when interpreting success of multidomain lifestyle interventions. However, several risk factors for dementia captured in the LIBRA, e.g., physical inactivity, did not change due to the intervention, possibly requiring more intensive interventions and support of participants in conducting the intervention.

